# The Impact of 45S5-Bioactive Glass on Synovial Cells in Knee Osteoarthritis—An In Vitro Study

**DOI:** 10.3390/ma16247594

**Published:** 2023-12-11

**Authors:** Hadrian Platzer, Max Marinescu, Qaisar Nawaz, Elena Tripel, Simone Gantz, Axel Horsch, Volker Daniel, Aldo R. Boccaccini, Sébastien Hagmann, Babak Moradi, Tobias Renkawitz, Fabian Westhauser

**Affiliations:** 1 Department of Orthopaedics, Heidelberg University Hospital, 69118 Heidelberg, Germany; hadrian.platzer@med.uni-heidelberg.de (H.P.);; 2 Institute of Biomaterials, University of Erlangen-Nuremberg, 91085 Erlangen, Germany; 3 Institute of Immunology, Heidelberg University Hospital, 69120 Heidelberg, Germany; 4 Department of Orthopedics and Trauma Surgery, University Hospital Kiel, 24105 Kiel, Germany

**Keywords:** osteoarthritis, synovial cells, 45S5-bioactive glass, MMP, cytokine, inflammation

## Abstract

Synovial inflammation in osteoarthritis (OA) is characterized by the release of cartilage-degrading enzymes and inflammatory cytokines. 45S5-bioactive glass (45S5-BG) can modulate inflammation processes; however, its influence on OA-associated inflammation has hardly been investigated. In this study, the effects of 45S5-BG on the release of cartilage-degrading metalloproteinases and cytokines from synovial membrane cells (SM) isolated from patients with knee OA was assessed in vitro. SM were cultivated as SM monocultures in the presence or absence of 45S5-BG. On day 1 (d1) and d7 (d7), the concentrations of Matrix Metalloproteinases (MMPs) and cytokines were assessed. In 45S5-BG-treated SM cultures, MMP9 concentration was significantly reduced at d1 and d7, whilst MMP13 was significantly increased at d7. Concentrations of interleukin (IL)-1B and C-C motif chemokine ligand 2 (CCL2) in 45S5-BG-treated SM cultures were significantly increased at both time points, as were interferon gamma (IFNG) and IL-6 at d7. Our data show an effect of 45S5-BG on SM activity, which was not clearly protective, anti-inflammatory, or pro-inflammatory. The influence of 45S5-BG on MMP release was more suggestive of a cartilage protective effect, but 45S5-BG also increased the release of pro-inflammatory cytokines. Further studies are needed to analyze the effect of BGs on OA inflammation, including the anti-inflammatory modification of BG compositions.

## 1. Introduction

Osteoarthritis (OA) is the most common joint disease worldwide [1], leading to a significant loss of quality of life through physical and social limitations [2,3]. In addition to the biomechanical cause of OA, biochemical processes, particularly inflammatory alterations, are thought to contribute substantially to cartilage damage and disease progression. Inflammation in the synovial membrane is a common feature in symptomatic OA patients [4] and is considered an independent cause of OA [5]. This synovial inflammation is characterized by the infiltration of inflammatory cells, such as macrophages and T cell subsets, that contribute to the production of inflammatory cytokines and metalloproteinases in OA joints [6,7,8,9,10,11,12]. This appears to throw metabolic and anabolic processes out of balance, which is considered critical in OA pathology. Matrix-metalloproteinases (MMPs), which can degrade major components of cartilage extracellular matrix appear to be key players in this pathological cartilage degrading process [13,14,15]. Although there is increasing evidence for inflammation as a cause of OA onset and disease progression, there is still no regulatory agency-approved disease-modifying osteoarthritis drug (DMOAD) [16].

The 45S5-bioactive glass (45S5-BG) composition, (wt%: 45SiO_2_-24.5Na_2_O-24.5CaO-6P_2_O_5_) has been successfully used as a bone substitute material, including but not limited to orthopedic applications mainly in bone defect treatment [17,18,19,20,21]. Beyond its application in orthopedic bone tissue engineering approaches [17], the possible fields of 45S5-BG application have significantly grown in the last years [22,23]: bioactive glasses (BGs) have not only been used to improve wound healing or to limit bacterial infections on devices, but they also showed promising properties in oncology [24,25,26,27,28,29]. It has been shown that the incorporation of ions with anti-inflammatory therapeutic potential in BGs does affect their biological performance, for example, by enhancing their osteogenic properties [30,31]. Recent research suggests that the 45S5-BG composition can modulate inflammatory processes, for example, towards an anti-inflammatory direction [32,33,34,35]. Other studies show that 45S5-BG might act pro-inflammatory when cultured in the presence of synovial cells: Bendall et al. investigated the effects of particulate 45S5-BG on synovial cells collected from a mixed patient collective suffering from either OA or rheumatoid arthritis (RA) [36]. They conclude that 45S5-BG might have a pro-inflammatory effect on synovial cells isolated from patients with OA; however, whilst the inflammatory response in synovial cells isolated from patients with RA was still increased in the presence of 45S5-BG, the extent of the inflammatory response decreased slightly with increasing BG concentration. Furthermore, the lowest concentration of 45S5-BG led to a decrease in the inflammatory response of synovial cells isolated from OA patients. Since both OA and RA are of an inflammatory character, Bendall et al. showed that 45S5-BG can modulate inflammation [36]. However, they only assessed the secretion of tumor necrosis factor-alpha (TNF) in their study, which is only one of several important inflammatory cytokines in OA development. The detailed anti-inflammatory potential of 45S5-BG has not been investigated in the field of OA research.

The overall aim of this study was to analyze the effects of 45S5-BG on synovial cell activity in patients with advanced knee OA. Specifically, we aimed to investigate the effects of 45S5-BG on the inflammatory profile of synovial membrane cells (SM) from patients with OA disease. To this end, we analyzed the effects of 45S5-BG microparticles on MMP and cytokine production of synovial cells from patients with advanced knee OA in vitro. Considering the literature, we focused on MMP1, MMP2, MMP3, MMP9, MMP13, interleukin (IL) 1B, IL4, IL6, IL10, IL-7A, tumor necrosis factor-alpha (TNF), interferon gamma (IFNG), and C-C motif chemokine ligand 2 (CCL2) [9,13,37,38,39,40,41,42,43].

## 2. Materials and Methods

### 2.1. Study Design Overview

**Figure 1:**
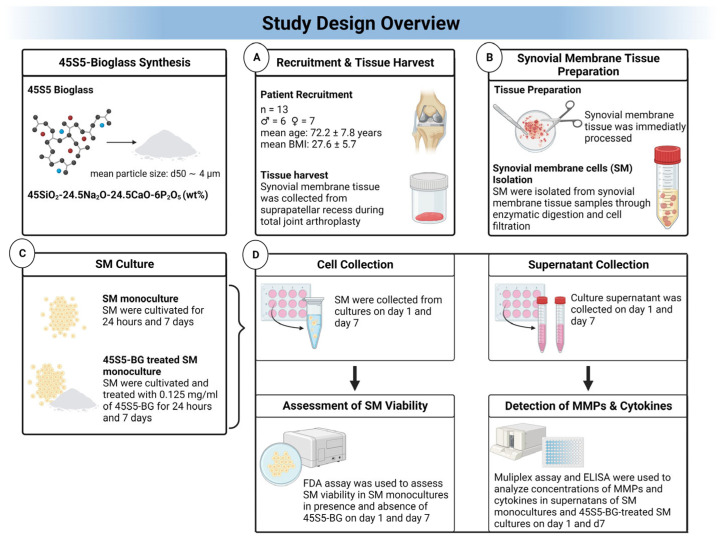
Study Design Overview. This graph provides an overview about the studies design. Abbreviations: Synovial membrane cells (SM), 45S5-Bioglass (45S5-BG), Fluorescein diacetate (FDA), Matrix-metalloproteinases (MMPs), Enzyme-linked Immunosorbent Assay (ELISA).

The study design overview is shown in Figure 1.

### 2.2. Study Population

The demographic parameters of the study population are shown in Table 1. In total, thirteen patients (7 female, 6 male) with a mean age of 72 ± 7.8 years, mean BMI of 27.6 ± 5.7 kg/m^2^, and advanced knee osteoarthritis were enrolled. OA was defined according to the American College of Rheumatology criteria. All patients had a Kellgren and Lawrence score (K&L score) of 3–4 and underwent total knee arthroplasty at the Clinic of Orthopedics, Heidelberg University Hospital. No patients included in this study showed signs of systemic inflammation (measured using C-reactive protein (CRP) and leukocytes prior to surgery) or reported joint trauma. Also, no patients reported a regular intake of nonsteroidal anti-inflammatory drugs (NSAID), any intake of disease-modifying drugs (DMARDS), corticosteroids or intraarticular injections of cortisone, or hyaluronic acid three months prior to surgery. The ethics committee of the University of Heidelberg (approval code: S-603/2021) approved this study. Informed consent from all patients was obtained prior to study enrolment.

### 2.3. Sample Collection

Synovial membrane tissue was harvested into sterile tubes with phosphate-buffered saline (PBS) from the suprapatellar recess during total joint arthroplasty by the performing surgeon. The tissue was immediately transported to our laboratory under sterile conditions for further processing (Figure 1A).

### 2.4. Bioactive Glass

Melt-derived 45S5-bioactive glass (BG) powder (Schott Vitryxx) with a mean particle size of d50 ~4 μm and a nominal composition of 45 SiO_2_, 24.5 Na_2_O, 24.5 CaO, and 6 P_2_O5 (in wt.%) was used. The used commercial 45S5-BG powder has been previously analyzed in terms of bioactivity; therefore, these experiments were not carried out within this study [44].

### 2.5. Synovial Membrane Tissue Preparation and Synovial Membrane Cell Isolation

Synovial membrane tissue was processed as described before [6]. After primary tissue processing, enzymatic digestion for 2 h and cell filtration to remove any debris and undigested tissue, isolated synovial membrane cells (SM) were washed with PBS and erythrocytes were lysed with lysis buffer (Figure 1B). The lysis buffer was prepared using 500 milliliters (mL) of two times distilled water, 0.5 g (g) of potassium bicarbonate ($KHCO_{3}$) (Carl Roth, Karlsruhe, Germany), 4.15 g of ammonium chloride (${NH}_{4}Cl$) (Carl Roth, Karlsruhe, Germany), and 18.5 milligrams (mg) of ethylenediaminetetraacetic acid (EDTA) (Sigma-Aldrich, St. Louis, MO, USA). The buffer was set to a pH-Value of 7.43 using hydrochloric acid (HCl). Synovial cells were counted using a MACSQuant^®^ Analyzer (Miltenyi Biotec GmbH, Bergisch Gladbach, Germany).

### 2.6. Cell Culture

SM were cultivated in a 12-well plate with Dulbecco’s modified Eagle’s medium (DMEM) supplemented with 10% fetal calf serum (FCS) (Bio&SELL GmbH, Nürnberg, Germany) and 5 milligrams per milliliter (mg/mL) penicillin/streptomycin (Biochrom, Berlin, Germany) with 450,000 synovial cells per well at 37 °C and 5% CO_2_. SM were cultured as SM monocultures in the presence or absence of 45S5-BG (Figure 1C). A total of 0.125 mg/mL of 45S5BG was established to be the highest non-toxic concentration for synovial cells (see 2.7 Cell Viability Assay/FDA). Culture supernatant was collected and stored at −80 °C for further analysis on day 1 (d1) and day 7 (d7) after initial cultivation.

### 2.7. Cell Viability Assay/FDA

Fluorescein diacetate (FDA; Sigma–Aldrich, St. Louis, MO, USA) was used to determine the cell viability of the cell cultures as described previously [45]. FDA is hydrolyzed into a green, fluorescent product by living cells with an active metabolism, which accumulates. The fluorescence signal correlates with the number of viable cells. After the removal of the culture supernatant, cells were washed with PBS and stained with 1 mL of FDA staining solution (1:50 dilution of FDA stock solution in PBS), with FDA stock solution consisting of 0.1 mg/mL FDA dissolved in acetone (Carl Roth, Karlsruhe, Germany), and incubated for 10 min at 37 °C in the dark. After incubation, FDA was removed, and cells were washed again with PBS, to remove any remaining FDA in the solution. Cells were then lyzed with 1% Triton X-100 buffer (Sigma–Aldrich, St. Louis, MO, USA). The fluorescence intensity was quantified at 535 nm for emission and 485 nm for excitation using a Wallac 1420 Victor 2 microplate reader (Perkin Elmer, Waltham, MA, USA).

In preliminary experiments, FDA cell viability staining was used to determine 45S5-BG-toxicity on SM (N = 6). SM derived from OA patients with the same inclusion and exclusion criteria from this study were cultivated as SM monocultures in the presence or absence of 45S5-BG at different concentrations (0.0625 mg/mL, 0.125 mg/mL, and 0.25 mg/mL). After 1 day and after 7 days, FDA cell viability staining was used to determine SM cell viability. The highest non-toxic concentration of 45S5-BG was determined to be 0.125 mg/mL. Higher concentrations of 45S5-BG in cell culture resulted in higher cell cytotoxicity, while lower concentrations would in theory result in lower effects.

### 2.8. ELISA and Multiplex Assay

Enzyme-linked Immunosorbent Assays were used to analyze the culture supernatant for concentration of MMP1 (Sigma–Aldrich, RAB0361), MMP2 (Sigma–Aldrich, RAB0365), MMP3 (Sigma–Aldrich, RAB0367), MMP9 (Sigma–Aldrich, RAB0372), MMP13 (Sigma–Aldrich, RAB0364), and IL-6 (R&D Systems, Minneapolis, MN, USA, DY206), according to the manufacturer’s instructions. The analyte detection sensitivities were determined to be 8 pg/mL for MMP1, 3500 pg/mL for MMP2, 300 pg/mL for MMP3, 10 pg/mL for MMP9, 6 pg/mL for MMP13, and 10 pg/mL for IL6. The photometric analysis was completed using an Autobio PHOmo Microplate Reader (Autobio Diagnostics Co., Ltd., Zhengzhou, China).

A Multiplex assay (Thermo Fisher Scientific, Dreieich, Germany) was used to measure concentrations of cytokines IFNG, IL1B, IL4, IL10, IL17A, TNF, and CCL2 in culture supernatants. Supernatants were applied undiluted according to the manufacturer’s instructions and the plate was analyzed using a Luminex 200 Instrument with xPONENT software (Version Build 4.2.1509.0; Luminex Corp., Austin, TX, USA). The ProcartaPlex high-sensitivity immunoassay used in this study provides high-sensitivity detection of cytokine concentrations in the range of fg/mL with a minimum bead count of 50.

### 2.9. Statistical Analysis

Quotients of analyte concentration to the corresponding FDA value were calculated and used for statistical comparison. Using IBM SPSS Statistics (Version 27; IBM, Mannheim, Germany), after performing a Kolmogorov–Smirnov Test to test data for normality, a paired test was used for normally distributed data. For non-parametric data, a Wilcoxon rank test was used. Graphs were designed with GraphPad Prism (Version 9; GraphPad Software, La Jolla, CA, USA). Results were considered statistically significant at a *p*-value of <0.05. *p*-values are marked as follows: * *p* < 0.05, ** *p* < 0.01, *** *p* < 0.001.

## 3. Results

### 3.1. The Impact of 45S5-BG on Synovial MMP Production

MMP3 was detected with the highest concentration of MMPs in both SM monocultures and 45S5-BG-treated cultures followed by MMP1, MMP9, and MMP2. MMP13 was detected with the lowest concentration by a factor of 1000 compared to the other MMPs.

**Figure 2:**
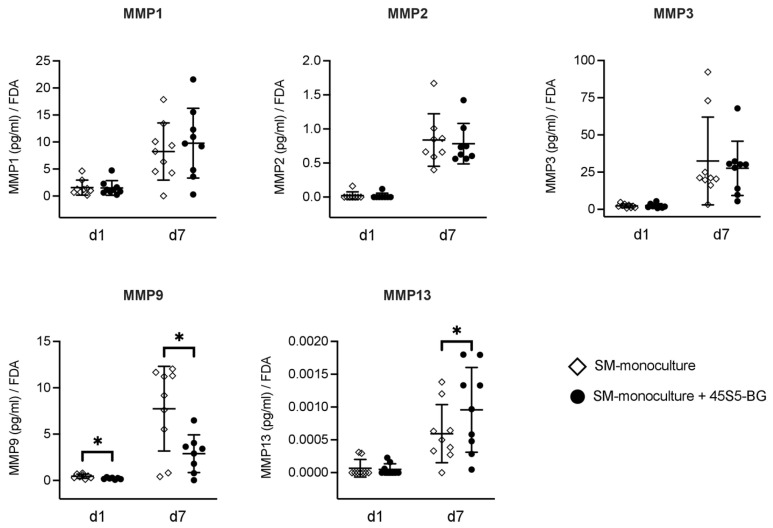
Quantitative analysis of Matrix-Metalloproteinase (MMP) concentrations in supernatants of synovial membrane cell (SM) monocultures in the presence or absence of 45S5-bioactive glass (45S5-BG). Enzyme-linked immunosorbent assays (ELISA) were used to analyze MMP enzyme concentration in culture supernatants at time points day 1 (d1) and day 7 (d7). Quotients of analyte concentration to the corresponding FDA value were calculated. Mean and standard deviation are plotted (N = 7–9). Significant differences are indicated by asterisks: * *p* < 0.05.

No significant differences in the concentrations of MMP1, MMP2, and MMP3 were detected between the supernatants of SM monocultures in the absence of 45S5-BG and 45S5-BG-treated SM cultures for both time points d1 and d7 (Figure 2). The concentration of MMP9 was significantly reduced in the supernatant of 45S5-BG-treated SM cultures when compared to SM monocultures in the absence of 45S5-BG for both time points d1 (*p* = 0.001) and d7 (*p* = 0.004) (Figure 2). At d7, MMP13 levels were significantly increased in the supernatant of the 45S5-BG-treated SM cultures compared to the SM monocultures in the absence of 45S5-BG (*p* = 0.007), with no significant differences at d1 (Figure 2).

### 3.2. The Impact of 45S5-BG on Synovial Cytokine Production

**Figure 3:**
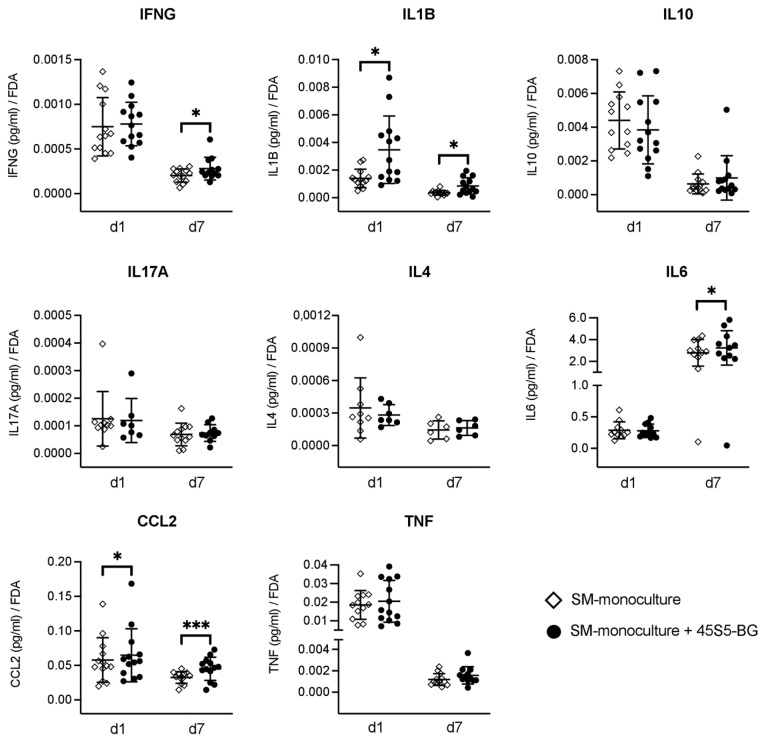
Quantitative analysis of cytokine concentrations in supernatants of synovial membrane cell (SM) monocultures in the presence or absence of 45S5-bioactive glass (45S5-BG). A multiplex assay was performed to measure cytokine concentrations at time points day 1 (d1) and day 7 (d7). Quotients of analyte concentration to the corresponding FDA value were calculated. Mean and standard deviation are plotted (N = 10–13, IL4 N = 6 with only low concentration on d1). Significant differences are indicated by asterisks: * *p* < 0.05.

No significant differences in concentrations between supernatants of SM monocultures in the absence of 45S5-BG and 45S5-BG-treated SM cultures were detected for IL4, IL10, IL17A, and TNF (Figure 3).

In the supernatant of 45S5-BG-treated SM cultures at both time points d1 and d7, the concentration of IL1B (*p* = 0.04, *p* = 0.02) and CCL2 (*p* = 0.046, *p* = 0.001) were significantly higher when compared to SM monocultures in the absence of 45S5-BG (Figure 3). At d7 in the supernatants of 45S5-BG-treated SM cultures, significantly higher concentrations of IFNG (*p* = 0.028) and IL6 (*p* = 0.05) were detected compared to SM monocultures in the absence of 45S5-BG with no significant differences at d1 (Figure 3).

## 4. Discussion

Local inflammatory processes are thought to play a critical role in OA pathology. In OA joints, increased production of cartilage degrading MMPs, and inflammatory cytokines appear to be associated with cartilage degradation and accelerated disease progression [6,9,15,46]. Synovial cells are considered a major source of such metalloproteinases and cytokines in OA joints [6,9,46]. Although several anti-inflammatory approaches have been tested, there is still no regulatory agency-approved disease-modifying osteoarthritis drug (DMOAD) [16].

The overall aim of this study was to analyze the effect of 45S5-BG, which has recently been reported to have anti-inflammatory potential [32,33,34,35], based on the production of cytokines (IL1B, IL4, IL6, IL10, IL17A, TNF, IFNG, and CCL2) and MMPs (MMP1, MMP2, MMP3, MMP9, and MMP13) from SM of patients with advanced knee OA in vitro.

It has previously been reported that BG particles have a concentration-dependent effect on the secretion of TNF in synovial cells isolated from OA patients. Low BG concentrations seem to decrease the TNF secretion of synovial cells while higher concentrations, comparable with the concentrations used in this analysis, might enhance TNF production [36]. In this study, however, 45S5-BG did not exhibit any significant effect on TNF secretion from SM. Thus, a potentially suppressive effect of 45S5-BG on TNF secretion from SM may have been masked in this study because of the 45S5-BG concentration used.

IL4 and IL10 are regarded as the main anti-inflammatory cytokines with a strong chondroprotective effect in OA pathophysiology [47]. In this study, IL4 and IL10 showed no significant changes between supernatants of SM monocultures in the absence of 45S5-BG and 45S5-BG-treated SM cultures. Also, in this study, no effect of 45S5-BG on the synovial production of inflammatory cytokine IL17A, which was shown to have cartilage destructive potential [48,49], was detected.

Whether 45S5-BG microparticles have any effect on these cytokines released from SM or if such effect might be concentration-dependent cannot be clearly stated and remains subject to further research.

A significant effect of 45S5-BG on synovial production of IL1B, IL6, IFNG, and CCL2 was detected. Elevated IL1B and IFNG levels in OA joints are thought to contribute to cartilage damage and OA disease progression by inducing chondrocyte apoptosis, upregulating cartilage degrading enzymes, and inhibiting extracellular matrix (ECM) component synthesis [39,40,41,50,51]. IL6, which is a major pro-inflammatory cytokine, was found to be increased in OA synovial fluid when compared to synovial fluid of control patients [52] and seems to have a pivotal role in OA pathology [53]. CCL2 concentration in synovial fluid has been associated with pain and disability in OA patients and has been shown to increase the release of MMP3 from chondrocytes inducing the degradation of cartilage matrix components in OA joints [42,43]. In this study, 45S5-BG resulted in increased production of IL1B, IL6, IFNG, and CCL2, suggesting a catabolic and pro-inflammatory rather than a protective and anti-inflammatory effect of 45S5-BG with respect to cytokine release from SM.

In the next step, to further determine the effect of 45S5-BG on synovial inflammatory processes, we analyzed the synovial production of several MMPs in supernatants of SM monocultures in the absence of 45S5-BG and 45S5-BG-treated SM cultures. All measured MMPs (MMP1, MMP2, MMP3, MMP9, and MMP13) were detected in supernatants of SM cultures in this study. This is consistent with the results of previous studies demonstrating such MMP production or expression in SM from OA patients [9,37], supporting the potential of SM to contribute substantially to the cartilage degradation process in OA pathology. MMP3 was detected with the highest concentration in this study, followed by MMP9, MMP1, and MMP13, which is also consistent with previous results from our group [6]. MMP1, also known as interstitial collagenase, MMP2 (gelatinase A), and MMP3 (stromelysin-1), seem to be associated with OA pathology by their potential to destruct the major components of cartilage extracellular matrix [13,54,55]. While 45S5-BG microparticles did not show any significant effect on synovial MMP1, MMP2, or MMP3 production, 45S5-BG significantly changed the production of MMP9 and MMP13, indicating the potential of 45S5-BG to influence synovial cell MMP production in general. MMP9, gelatinase B, which is capable of digesting denatured collagen fibrils [56], appears to be induced mainly by macrophages and T cells in synovial tissue [6]. MMP9 concentration in OA joints is increased, suggesting that MMP9 plays an important role in OA pathology [38]. In this study, 45S5-BG significantly reduced MMP9 production in SM cultures at both time points, d1 and d7 (Figure 2), suggesting that 45S5-BG microparticles can prevent the cartilage degradation process through its suppressive impact on synovial MMP9 production. Similarly, Li et al. were able to effectively reduce MMP9 expression and activity in human umbilical vein endothelial cells and fibroblasts, using 45S5-BG [57]. In contrast with the suppressive effect of 45S5-BG on synovial MMP9 production, a significantly higher concentration of collagenase MMP13 was detected in supernatants of 45S5-BG-treated cultures at d7 compared to SM monocultures in absence of 45S5-BG. MMP13 is mainly produced in chondrocytes; it specifically degrades collagen type II and increased activity of MMP13 is thought to contribute substantially to the destruction of ECM in OA pathology [13,58,59].

Considering that cartilage and not SM is thought to be the main source of MMP13 [13,59] and that MMP13 was detected at by far the lowest concentration, at least 1000-fold lower than all other detected MMPs, the increase in synovial MMP13 production might be negligible in contrast to results concerning MMP9. Thus, the effects of 45S5-BG on synovial MMP production detected in this study are more suggestive of an anti-inflammatory and cartilage-protective effect.

The observed scatter of data values could not be explained in this study, which may be due to the limited number of patients included in the study. Therefore, further studies with a larger number of patients should be conducted. Furthermore, the concentration of 45S5-BG seems to play a role in the inflammatory response of synovial cells as well: the work of Bendall et al. shows that the secretion of TNF in synovial cells derived from patients suffering from RA slightly decreases with increasing presence of 45S5-BG [36]. The effects of BG concentrations on the inflammatory response were not evaluated in this study since we aimed to use the highest, non-toxic concentration of 45S5-BG for the downstream experiments. It is well known that 45S5-BG can exhibit cytotoxic effects under in vitro conditions and the limitation of these effects is crucial in order to assess the biological effects of the glasses [60]. However, since the lowest concentration of 45S5-BG particles (1 µg/mL) in the study of Bendall et al. slightly decreased the TNF secretion in synovial cells from OA patients [36], the effects of 45S5-BG might be of anti-inflammatory character when 45S5-BG is used in lower rather than the highest non-toxic concentrations like in this study. Therefore, future studies should consider the concentration-dependent effects of 45S5-BG.

## 5. Conclusions

In contrast to the demonstrated effect of 45S5-BG on synovial MMP release, which can be assumed to be more cartilage protective due to the suppressive effect of 45S5-BG on synovial MMP9 production, the results of this in vitro study rather show an inflammatory effect of 45S5-BG on synovial cells with respect to synovial cytokine release.

Nevertheless, this in vitro study clearly demonstrates that 45S5-BG can affect MMP and cytokine release of synovial cells from patients with advanced knee OA. The fact that the chemical composition of 45S5-BG particles can be modified, for example by the introduction of ions exhibiting anti-inflammatory activity, highlights the therapeutic potential of 45S5-BG, in particular, or BGs in general on inflammatory diseases, such as OA. However, to clarify the anti-inflammatory potential of 45S5-BG on OA joints, further studies are needed to investigate the effect of 45S5-BG not only on synovial tissue but also on cartilage. Further, the effect of 45S5-BG, including anti-inflammatory components on OA inflammation processes, needs to be investigated.

## Figures and Tables

**Table 1:** Demographic and clinical parameters of the study population are displayed. Data are presented as mean ± standard deviation or as number (%). BMI = body mass index.

	Total Study Population
Number of patients	13
Gender: male	6 (46.2%)
Gender: female	7 (53.8%)
Age at surgery, years	72.2 ± 7.8
BMI, kg/m^2^	27.6 ± 5.68
Leukocytes, cells/nL	6.2 ± 1.5
C-reactive protein, mg/L	2.1 ± 3.4

## Data Availability

The data presented in this study are available on request from the corresponding author.
